# Delamination and Manufacturing Defects in Natural Fiber-Reinforced Hybrid Composite: A Review

**DOI:** 10.3390/polym13081323

**Published:** 2021-04-18

**Authors:** M. J. Suriani, Hannah Zalifah Rapi, R. A. Ilyas, Michal Petrů, S. M. Sapuan

**Affiliations:** 1 Faculty of Ocean Engineering Technology and Informatics, Universiti Malaysia Terengganu, Kuala Nerus, Terengganu 21030, Malaysia; hnazara98@gmail.com; 2 Marine Materials Research Group, Faculty of Ocean Engineering Technology and Informatics, Universiti Malaysia Terengganu, Kuala Nerus, Terengganu 21030, Malaysia; 3 Faculty of Engineering, School of Chemical and Energy Engineering, Universiti Teknologi Malaysia, UTM Johor Bahru, Skudai, Johor 81310, Malaysia; 4 Centre for Advanced Composite Materials, Universiti Teknologi Malaysia, UTM Johor Bahru, Skudai, Johor 81310, Malaysia; 5 Faculty of Mechanical Engineering, Technical University of Liberec, Studentská 2, 461 17 Liberec, Czech Republic; michal.petru@tul.cz; 6 Laboratory of Biocomposite Technology, Institute of Tropical Forestry and Forest Products (INTROP), Universiti Putra Malaysia, UPM Serdang, Seri Kembangan, Selangor 43400, Malaysia; sapuan@upm.edu.my; 7 Advanced Engineering Materials and Composites Research Centre (AEMC), Department of Mechanical and Manufacturing Engineering, Faculty of Engineering, Universiti Putra Malaysia, UPM Serdang, Seri Kembangan, Selangor 43400, Malaysia

**Keywords:** delamination, manufacturing defects, mechanical properties, hybrid composite, natural fiber-reinforced

## Abstract

In recent years, most boat fabrication companies use 100% synthetic fiber-reinforced composite materials, due to their high performance of mechanical properties. In the new trend of research on the fabrication of boat structure using natural fiber hybrid with kevlar/fiberglass-reinforced composite, the result of tensile, bending, and impact strength showed that glass fiber-reinforced polyester composite gave high strength with increasing glass fiber contents. At some point, realizing the cost of synthetic fiber is getting higher, researchers today have started to use natural fibers that are seen as a more cost-effective option. Natural fibers, however, have some disadvantages, such as high moisture absorption, due to repelling nature; low wettability; low thermal stability; and quality variation, which lead to the degradation of composite properties. In recent times, hybridization is recommended by most researchers as a solution to natural fiber’s weaknesses and to reduce the use of synthetic fibers that are not environmentally friendly. In addition, hybrid composite has its own special advantages, i.e., balanced strength and stiffness, reduced weight and cost, improved fatigue resistance and fracture toughness, and improved impact resistance. The synthetic–nature fiber hybrid composites are used in a variety of applications as a modern material that has attracted most manufacturing industries’ attention to shift to using the hybrid composite. Some of the previous studies stated that delamination and manufacturing had influenced the performance of the hybrid composites. In order to expand the use of natural fiber as a successful reinforcement in hybrid composite, the factor that affects the manufacturing defects needs to be investigated. In this review paper, a compilation of the reviews on the delamination and a few common manufacturing defect types illustrating the overview of the impact on the mechanical properties encountered by most of the composite manufacturing industries are presented.

## 1. Introduction

In recent years, most boat fabrication companies utilize 100% synthetic fiber-reinforced composite material due to the high performance of mechanical properties. This is also as claimed by El-wazery, El-elamy, and Zoalfakar [1]. In their research on the fabrication of boat structure using kevlar/fiberglass-reinforced composite, the result of tensile, bending, and impact strength showed that glass fiber-reinforced polyester composite gave high strength with increasing glass fiber contents. However, fiberglass is expensive and, in terms of environmental concern, it gives a high impact on the ecosystem as well as crosses over the area of occupational health and safety concern.

Today, the use of natural fibers has become a trend in boat manufacturing and other equipment, due to their light weight; good relative mechanical properties [2]; more important factors, such as being eco-friendly and sustainable materials [3]; as well as lower cost, compared to fiberglass [2]. Since then, the shifting of interest by the composite manufacturing industries to hybrid composite has taken place. Natural fiber-reinforced hybrid usually indicates a thermosetting of natural fiber to a combination of two or more reinforced elements in a single matrix or a mixture of different matrices merged with a single reinforced element. Many of the composites used today are at the forefront of material science, with performance and cost suitable for ultrademanding applications, such as in maritime sectors.

Hybridization is recommended by most researchers as a solution to natural fiber weaknesses and to reduce the use of synthetic fibers that are not environmentally friendly. Hybridization is a process in which natural fiber and artificial fiber are combined in a composite [4,5,6,7,8,9,10,11,12]. The modern materials used in a broad variety of applications are synthetic–natural fiber hybrid composites. Natural fibers, however, have some disadvantages, such as high moisture absorption, due to their repelling nature; low thermal steadiness; low wettability; and variation in quality [13]. Earlier studies surmised that the key factor restricting the mechanical properties of natural fiber-reinforced polymer composites was the chemical incompatibility between plant fiber’s molecules and thermoplastic/thermoset molecules [14,15]. Therefore, this review paper compiles the review on the delamination and the common manufacturing defects in natural fiber-reinforced hybrid polymer composites that influences the performance of the composites.

## 2. Composite Delamination

One of the most prevalent failure forms of composite materials is delamination. Due to imperfections during the manufacturing process or the effects of external factors during the working life of composite laminates, i.e., the impact of foreign items, the phenomenon of delamination may occur [16]. This failure also happens due to the high interlaminar stresses that are connected, typically, to the lowest through-thickness strength. This is caused by the fibers that lie in the laminate plane that do not reinforce the thickness, so the composite must rely on the nearly weak matrix to transport loads in that direction [17].

Delamination is a type of layer deformation in laminated composite materials, and it is due to continuous stress and pressure on the material. This form of failure can result in faulty performance during the use of these materials. The inadequate curing methods form irregular pressure on the different areas, generating areas of delamination. The formation of these delaminated areas in composite materials can significantly reduce the composite’s strength during compressive loading. This is because of the buckling effect of the laminated structure.

According to Imran et al. [18], there are several types of models of failure, and interlaminar delamination is one of the most common models. The structural stability of the thin-walled laminated fiber-reinforced polymer composite materials is a serious problem, when it is associated with delamination, as buckling in the structure of composite will affect mechanical and structural properties. Therefore, this failure model should be considered in the design process before starting the fabrication process.

**Figure 1:**
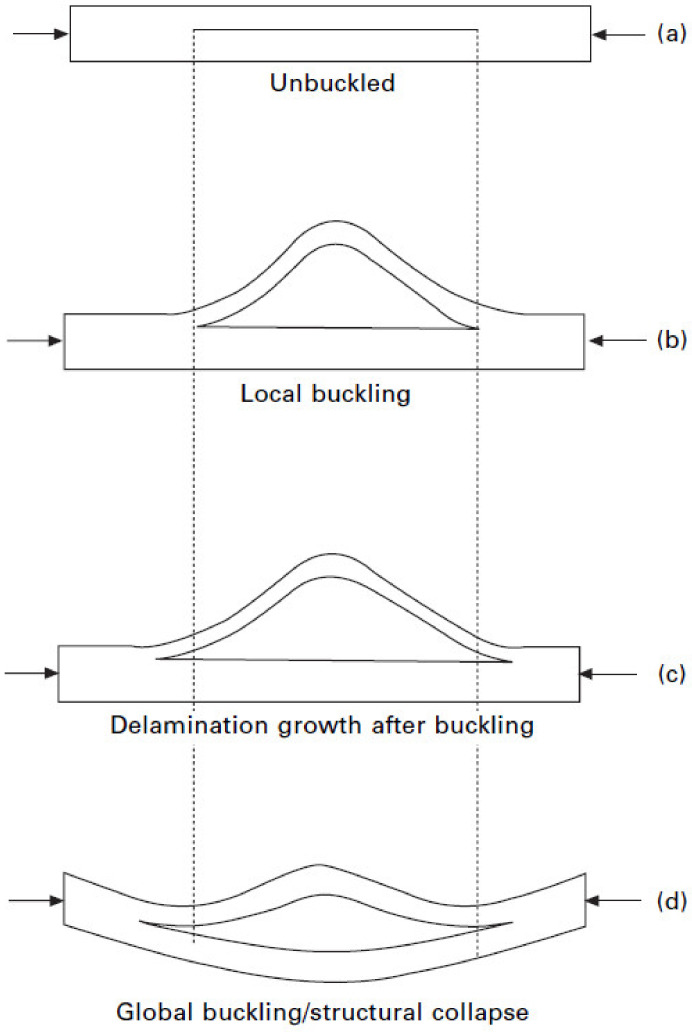
Compression behavior of delaminated composite panels. (**a**) unbuckled, (**b**) local buckling, (**c**) delamination growth after buckling, (**d**) global buckling/structural collapse.

It can be observed from Figure 1 that, when a delaminated composite plate faces in-plane compression, local or global buckling of the delaminated region may occur. In several cases, mixed-mode buckling also can happen, in which both local and global buckling can occur simultaneously in the laminated composite. Therefore, it is recommended to analyze the delamination of laminated composites after the buckling [18].

During the compression process, the delaminated composite plate possesses the poor capability to resist compressive loads. According to Hwang et al. [19], the reduction in this ability is dependent on the properties of the delamination within the composite, such as position, area, and shape of the delamination. Nevertheless, if the delamination does not develop within the composite in a short period, the buckling of the delaminated composite plate may not represent the immediate failure. It was investigated that the growth of delamination within composites always takes place after buckling. Therefore, even after buckling, the delaminated composite plate continues to suffer from an increasing load until the delamination develops. The key growing potential of the delaminated composite platform reflects the ability to withstand compressive loads. Hence, understanding the impact of delamination on the buckling and postbuckling behaviors is important in order to properly design the laminated composites and to safely use fiber-reinforced composite materials [19].

In Figure 1, the steps leading to a composite panel’s delaminated failure under compression are displayed: condition of unbuckled structure (Figure 1a), local buckling (Figure 1b), delamination growth (Figure 1c), and structural collapse (Figure 1d). For specific structural details, including the bolted joints, the tensile loading condition can make a local compressive stress state, critical for delamination growth, near the hole. Moreover, delamination onset takes place in the bearing plane when joints fail, following the bearing failure mode. These delamination failures tend to develop under tensile–compression fatigue or tensile loading conditions.

Instead of the delamination in a composite material that can occur during the production process, the composite material can also experience many different defects, which are also influenced by the manufacturing methods, also known as manufacturing defects. According to Potter et al. [20], the term “defect” refers to an abnormality in a material or structure that causes it to depart from its specification as described during the design process. Generally, defect detection can be very challenging and difficult to predict, especially on the effect of their structural properties [21]. The manufacturing defects in fiber-reinforced composites involve misalignment, waviness, and sometimes fiber breakage, fiber or matrix debonding, delamination, and void formation in the matrix of composite materials [22].

In theory, such departures from specification can be resolved by strict adherence to the manufacturing process, leading to the idea that defects can be avoided. Finally, the defects are known to be introduced in the manufacturing process.

## 3. Classification of Manufacturing Defects

The manufacturing of composite materials can be done by a broad range of methods. For instance, the process of polymer matrix composites can be performed by using the “lay-up” method, one of the most common techniques used in the industry that is also called the wet lay-up or hand lay-up method [23]. It is the simplest process, where each ply is stacked layer-by-layer depending on the desired thickness, which is handled by hand [24]. In addition, the compression molding, liquid molding, injection molding, and resin infusion processes can also be used in composite materials. During the manufacturing process, the composite materials usually experience a few defects that affect the fiber surfaces or between the layers, known as delamination [25]. This defect can be classified into five classes, which later will lead to composite failure.

### 3.1. Voids

In the manufacturing process, porosity, which is also known as void, is one of the damaging defects that arises, and, in structural composites, it plays a major role in mechanical performance [26,27]. Commonly, voids can be described as a phenomenon when there are air bubbles trapped in the matrix while the composite undergoes fabrication, which is caused by several factors [23], such as curing pressure, resin system, and environmental condition. It also becomes a common defect that can easily be introduced into the material during the manufacturing process [28].

Because of the heterogeneity in thermodynamic and rheological phenomena that exist in these systems, during the processing of fiber-reinforced composites, the creation and growth of voids for all manufacturing techniques are different. In liquid composite molding, for example, void formation and evolution are commonly studied, focusing more on the voidage occurrence in the final process in autoclave curing, rather than the existence of voids during the process [26]. In the process of liquid composite molding, there are many reasons for the formation of voids. One of the main causes is the entrapment of mechanical air during resin flow [29]; other causes include the gas produced due to chemical reactions during curing [30], dissolved gases in the resin, and nucleation [31]. The air trapping is primarily due to the inhomogeneous nature of the fiber, resulting in the fiber preforms’ nonuniform permeability, which induces local variation in resin velocity. The capillary effect is aggravated by this local velocity variation, prevailing at the microscale [32].

### 3.2. Resin-Rich Zones

**Figure 2:**
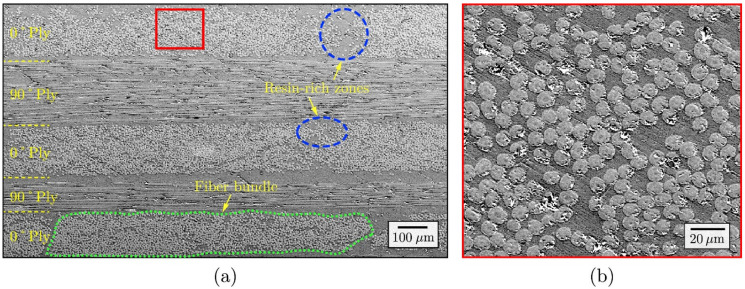
SEM micro-image of a cross-ply CFRP microstructure with embedded oval-shaped fibers: (**a**) arrangement of fibers in each ply, displaying fiber bundles and resin-rich zones; (**b**) larger view of the red inbox shown in Figure 2a. Reproduced with copyright permission from Ahmadian et al. [38].

The common phenomenon in the liquid composite molding process is the resin-rich zones that cause unwanted residual stress, deformation, and part-to-part variations [33,34,35]. The resin-rich zones are formed during the resin transfer in the molding process [36]. During mold closure, the dry fiber preform is compressed in the resin transfer molding procedures. This compression pulls the fibers close at the corner radius for an angled portion, and a gap is formed between the fiber preform and the surface of the mold. A resin-rich zone is formed after the resin infusion [33]. In a study conducted by Holmberg et al. [37] on the manufacturing and performance of RTM U-beams, as the molds closed, the reinforcement tended to pull tight around corners, leaving a resin-rich area. Beam failure was caused by delamination in the radii. From this result, they concluded that the variations in the fiber content and fiber misalignment were of minor importance if the void content was low. In order to diminish preform defects, the void content within the composites had to be reduced by an appropriate level of vacuum assistance. At high fiber contents, the reinforcement easily wrinkled during preforming and/or mold closure. A study performed by Ahmadian et al. [38] on the effect of resin-rich zones on the failure response of carbon fiber reinforced polymers (CFRP) revealed that there were notable impacts on the failure response of statistical volume elements (SVE) by utilizing different boundary conditions and changing the compressive load direction. They concluded that the existence of large resin-rich zones in the matrix would lead to a strength reduction under tension and an increase in strength under compression. The SEM image of the CFRP microstructure studied in work done by Ahmadian et al. [38] is shown in Figure 2. It can be observed that the there were large resin-rich zones formed within the composites. This might be due to the lack of perfect integration between these carbon fibers during the fabrication process of resin infusion. In addition, it can be seen that smaller resin-rich zones were also formed within each bundle and, in particular, near their top/bottom edges. This was because of the carbon fibers’ relocation during the curing process.

A study was conducted by Idress et al. [39] on the effect of resin-rich layers on mechanical properties of 3D printed woven fiber-reinforced composites to determine the effects of resin-rich layer (RRL) thickness on mechanical properties of the composites. In their experiment, laminated composites were fabricated with controlled RRL thickness in the range of 0–200 µm and further tested for Mode I and Mode II interlaminar properties, short beam shear, flexure, and tensile. They concluded that RRL did not display any improvement in-plane or out-of-plane performance for the chosen materials. In addition, the plastic zone size was the key resin property as the trends in interlaminar toughness and strength were exhibited to strongly depend on the properties of the resin.

### 3.3. Pocket of Undispersed Crosslinker

Pockets of undispersed crosslinker can be caused by incomplete curing, curing agent’s distribution, or premature curing [40]. It is necessary to consider the rheological and mechanical properties of the adhesive/epoxy before checking the properties of composite structures. Changes in the adhesive due to incomplete curing can influence the adhesion test results [41]. The undercuring of thermoset composites leads to lower performance properties in the final product.

Glass fiber-reinforced polymers are normally cured at a “low” temperature (between 60 °C and 100 °C) and postcured at a higher temperature of about 150 °C or more to complete the curing and to achieve the maximum possible crosslinking rate and temperature of glass transition [42,43]. According to several studies, in order to increase the modulus and strength of both the polymer and the composite, as well as to reduce the residual stresses, postcuring is also required. However, a postcure can also lead to the thermo-oxidation of the resin. In addition, natural fibers could be degraded by certain curing and postcuring conditions, and their mechanical properties are mainly dependent on their water content [44,45,46]. Postcuring at high temperatures is likely to change the fibers’ water content and, thus, modify their mechanical behavior.

### 3.4. Misaligned Fibers

**Figure 3:**
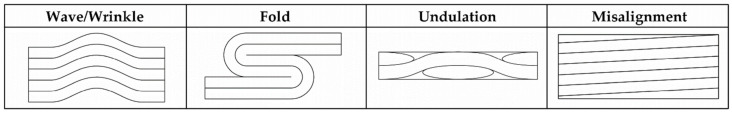
Fiber orientation of wave/wrinkle, fold, undulation, and misalignment. Reproduced with copyright permission from Thor et al. [47].

Fiber defects include misalignment, wrinkles, waviness, folds, undulation, and brokenness. Currently, there is no unanimously accepted terminology and consistent use for the differentiation between waves, wrinkles, folds, undulations, and misalignments, as illustrated in Figure 3. However, according to Thor et al. [47], the definition is constructed for the sake of clarity to the reader. The effect of manufacturing in polymer composite parts that results in decreased mechanical performance is usually due to the ply/fiber waviness or wrinkling conditions. Fiber waviness is known as a wave-formed ply and/or fiber deviation from a straight alignment in a unidirectional laminate. This phenomenon might be due to the detrimental manufacturing effect that generally occurs during consolidation/curing, infiltration, and/or draping process steps. Buckles or fiber buckling is referred to as out-of-plane fiber waviness, which happens due to stability issues when the ply is loaded under compression.

**Figure 4:**
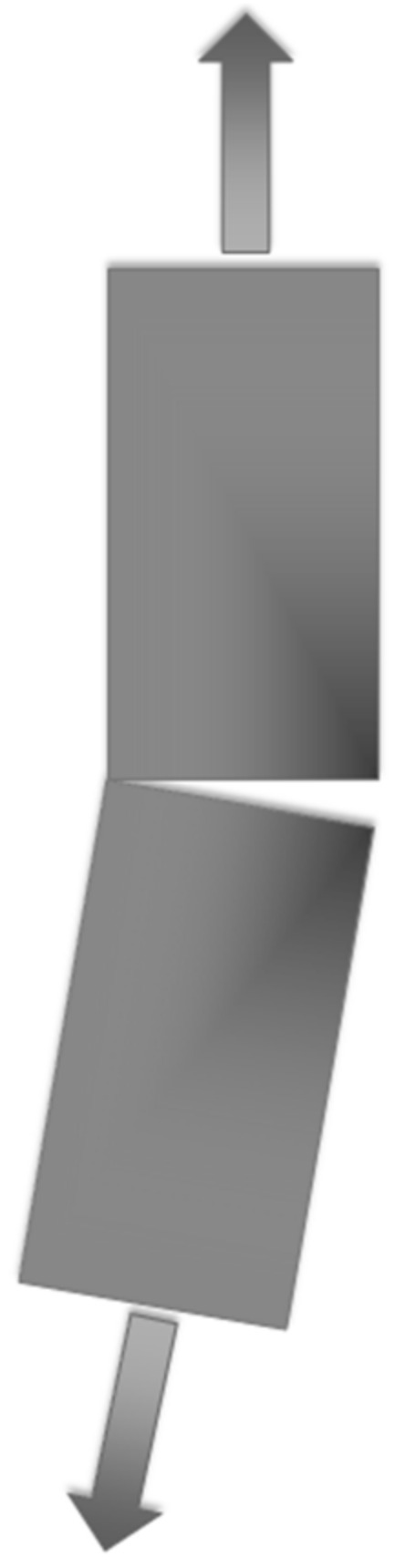
Stress distribution due to misaligned fibers.

In the world of fiber reinforced polymer composite (FRPC), alignment is a very important aspect. Unlike metals, which are isotropic in nature, the properties of FRPC vary significantly, according to the directionality of the specimen’s fibers. Alongside diminished reported strengths, misaligned machining also results in an uneven stress distribution among the remaining fibers, leading to premature failures in the high-stress region, as can be observed in Figure 4.

**Figure 5:**
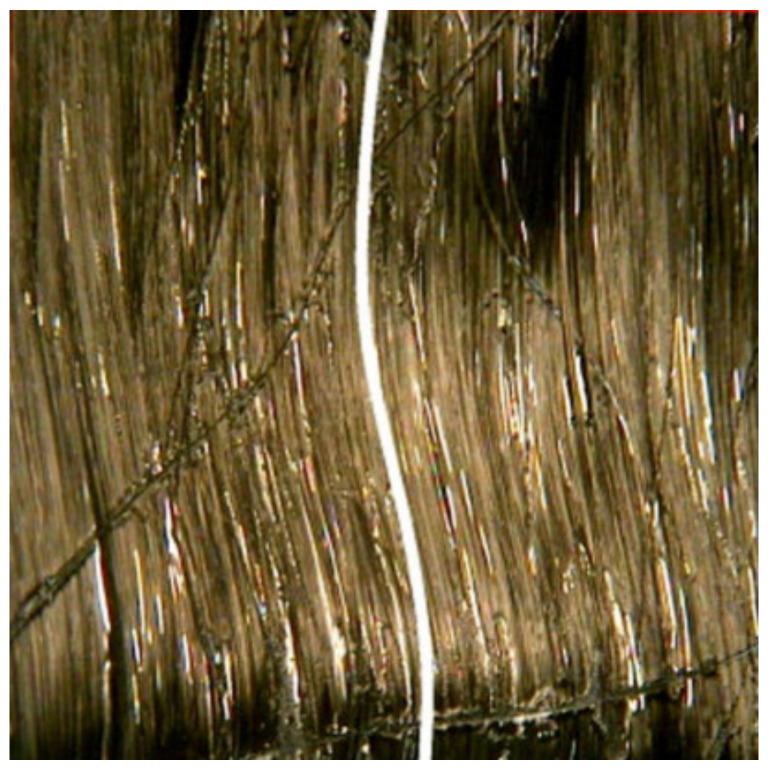
Level of fiber misalignment in a typical as-delivered unidirectional prepreg. Reproduced with copyright permission from Potter et al. [20].

**Figure 6:**
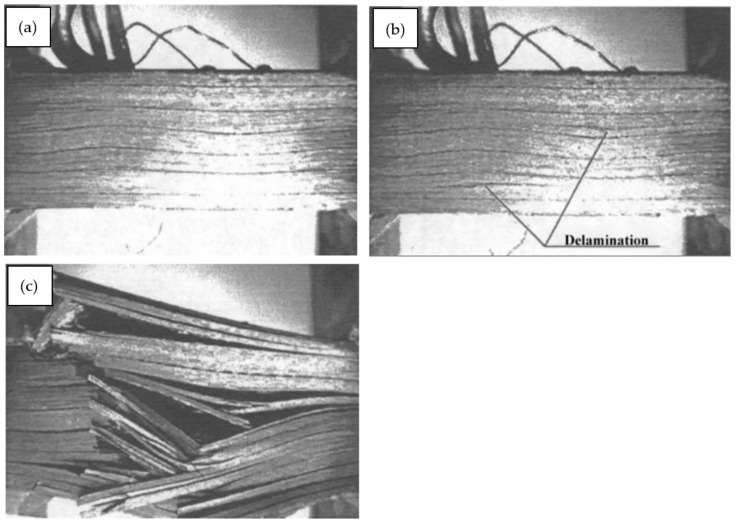
Illustration of the fiber waviness effect on failure under axial compression of a unidirectional carbon/epoxy composite. (**a**) Graded waviness of fibers before loading; (**b**): occurrence of delamination under compression; (**c**) final failure. Reproduced with copyright permission from Hsiao et al. [48].

Furthermore, in compression testing, any sources of misalignment can induce buckling, producing invalid results, regardless of the specimen appearing to fail by a valid failure mode. Additionally, in composites where fibers are considered straight, parallel, and oriented in planned directions, variations due to misalignment and waviness can decrease initial properties, particularly compression strength and rigidity, leading to decreases in aircraft design limit load and ultimate load capacity design in servicing capacity. The degree of misalignment observed in a standard as-delivered, unidirectional prepreg is shown in Figure 5, and the presence of delamination (separation of individual layers that were purposely rendered with wavy fibers) and final failure in axial compression are shown in Figure 6 [48]. The analysis of the impact of fiber waviness in axial compression unidirectional composites showed that, due to this type of defect, the stiffness and strength had decreased rigorously. Stress analysis and experimental observations demonstrated that delamination and subsequent failure were responsible for the interlaminar shear stress produced due to fiber waviness [48]. Therefore, in order to reduce misaligned fibers, Parlevliet et al. [49] and Baran et al. [50] reported the exclusive findings of composite materials’ residual stresses. An approach used to avoid the occurrence of ply wrinkling during curing was to maintain the thickness of the laminate under certain limits to reduce exothermal heat generation. Generally, the curing must be conducted carefully with controlled increments of temperature to reduce variations in thermal expansions. The probability of wrinkling could also be minimized by employing low forming speeds to ease the blank’s deformation by producing smaller resistance to interply and intraply shearing [51,52]. The wrinkling tendency is sourced from a so-called drape run-out that can be considerably reduced in the case of the forming layers that have the size and shape at the edges that result in as little extra material as possible [53]. Apart from that, it is recommended for preconsolidation to be perfromed using a vacuum bag at every four to five layers; this is essential to enhance the laminate quality, as well as minimize the risk of fiber waviness occurrence [54]. Better pressure distribution is achievable using rubber pads in a rubber-die molding process [53,55]. The method is similar to matched-die molding; however, it is more cost-effective, due to considerably lower mold costs, in addition to the risk reduction of the possibility for wrinkles via a more well-distributed pressure.

### 3.5. Region Where Resin Has Poorly Wetted the Fiber

The key issue of natural fibers in composites is the low compatibility between fiber and matrix and the relatively high absorption of moisture [56,57]. According to Xue Li [58], chemical treatments also improved interface adhesion between the fiber and the matrix and reduced the absorption of water by fibers. Chemical treatments should also be taken into account when altering the properties of natural fibers [58,59,60,61,62,63,64,65]. Additionally, cellulose, hemicellulose, lignin, pectin, waxes, and water-soluble substances constitute the components of natural fibers [66,67,68,69,70,71,72,73]. Table 1 indicates the composition of selected natural fibers. The natural fibers’ chemical composition and cell structures are quite complicated, as they differ in plant parts and origins. Each fiber is a composite by nature, in which rigid cellulose microfibrils are reinforced in the amorphous matrix composed of hemicellulose and lignin. Therefore, natural fibers can also be referred to as cellulosic or lignocellulosic fibers [74]. The mechanical, thermal, and physical properties of the natural fibers are different from one another, as they depend on their cellulose crystallinity [75]. Moreover, the chemical compositions of plant fibers are different, depending on the types of fiber. The main components of natural fibers are cellulose (30–80%), hemicellulose (7–40%), and lignin (3–33%), as shown in Table 1 [74,76,77]. Cellulose is an important structural component of the primary cell wall that surrounds the natural fibers, providing strength to plant cells, leaves, branches, and stems [78,79,80,81]. Cellulose is a semicrystalline polysaccharide consisting of units of d-glucopyranose interconnected by b-(1-4)-glucoside bonds [82,83,84,85]. However, in some cases when these natural fibers were used to reinforce hydrophobic matrices, a significant amount of hydroxyl groups in cellulose gave hydrophilic properties to natural fiber that resulted in very poor interface and resistance to moisture absorption [86].

Chemical treatments should also be considered when modifying the properties of natural fibers. Some compounds are known to promote adhesion by chemically coupling the adhesive to the substance, such as sodium hydroxide, silane, acetic acid, acrylic acid, maleate coupling agents, isocyanates, potassium permanganate, peroxide, etc. However, most chemical treatments have achieved various levels of success in improving fibers’ efficiency, fitness, and fiber–matrix adhesion in reinforced natural fibers [58].

## 4. Impact of Delamination and Manufacturing Defects

The difficulties associated with the manufacturing of laminated composites of carbon/glass fiber-reinforced epoxy are primarily associated with the presence of defects, such as voids, regions rich in resin, and misalignment of fibers. Those defects can lead to a serious impact on hybrid composites’ efficiency and provide an undesired variance in the mechanical properties resulting from them [104,105,106]. By reducing defects, several researchers have attempted to optimize the manufacturing process of composites [107,108]. In practice, removing all defects and creating a perfect composite material part is impossible to be done.

In most applications of composite materials, the existence of voids is undesirable, with the void content usually limited to below 5% [105]. However, even a void content of 1% is known to be inappropriate in some aerospace applications [109]. Several studies also have, so far, examined the relationship between void content and composite material strength, concluding that matrix-dominated properties, such as flexural and compressive strength, were more affected by voids than fiber-dominated properties, such as longitudinal tensile strength [110]. According to Mehdikhani et al. [26], the increase of 1% void content can lead to a decrease in tensile strength of 10% to 20%, 10% for flexural strength, and 5% to 10% for interlaminar shear strength [26]. In particular, flexural strength and modulus are extremely sensitive to void content [104,109]; thus, an important parameter in the design of composite materials is to consider the effect of matrix voids on the mechanical properties.

The key role of the matrix in fiber-reinforced composite material is to pass load between fibers and preserve the fibers under compressive load [111,112,113,114]. Therefore, when voids occur within the matrix, they are greatly influenced by the transverse and shear moduli and strengths, as well as the longitudinal compressive strength [110], since they are highly dependent on matrix properties and are known as “matrix-dominated properties.” The longitudinal tensile modulus and power, in comparison, are primarily influenced by the properties of the fiber and are, therefore, known as “fiber-dominated properties” and are, therefore, not significantly affected by the presence of voids.

### 4.1. Mechanical Properties of Hybrid Composite

Notably, in their products, most electrical and electronic industries use polymer matrix composites, and most applications use hybrid polymer matrix composites, due to their outstanding mechanical properties, compared to traditional polymer composites (polymer composites with single reinforcement that is either synthetic or natural). Natural and synthetic fibers are the two reinforcements widely used in polymer matrices for polymer composite manufacturing.

A review conducted by Azammi et al. [115] on the types of fiber and fiber loading demonstrated that these factors gave a huge effect on polymer composites. This was due to a good relationship between fibers with mechanical properties of the polymer composites. There are a lot of studies performed on the effect of fiber loading that led to the improvement of tensile strength [116,117,118]. It was reported that the optimum fiber loading for kenaf/thermoplastic polyurethane composites was 30% [119]. Other studies on reinforcement of kenaf fiber and phenol-formaldehyde (KF/PF) composites revealed that kenaf fiber loading of up to 43% exhibited the best tensile strength for the composites [120]. In addition, it can be observed from Table 2 that the highest tensile strength was obtained from pineapple fiber, with the values of 413–1627 MPa. This data can be correlated with Table 1, in which the mechanical, thermal, and physical properties of natural fibers are different from one another, as they depend on their cellulose contents. Moreover, Xiao et al. [121] had developed a fractal model for capillary flow through a single tortuous capillary with roughened surfaces in fibrous porous media. They found that the imbibition height and imbibition mass of the capillary had decreased with increasing relative roughness. The fractal theory is a very important tool that can be used to investigate the physical and mechanical properties of fiber reinforced composite material. Natural fibers are primarily cellulosic materials extracted from plants, i.e., bamboo, kenaf, abaca, coconut, sisal, and banana, while glass and carbon fibers are the two commonly used synthetic fibers as reinforcements in polymer matrix composites. Table 2 shows the mechanical and physical properties of different synthetic and natural fibers. Natural fibers are biodegradable [122], eco-friendly [123], cheap [124], and lighter [125] than most synthetic fibers. However, the mechanical strength of synthetic fibers is much greater than that of most natural fibers. Boopalan [126] reported that banana-reinforced epoxy composites possessed greater tensile and impact strengths than hybrid jute- and banana-reinforced epoxy composites, while hybrid composites had greater flexural strength than banana–epoxy composites. It can be concluded from this study that, in order to infuse desirable mechanical properties into polymer composites, hybridization is necessary.

Many studies reporting the effect of loading of synthetic fiber on the mechanical properties of natural fiber-reinforced thermosets polymer composites. It was revealed that the mechanical properties of these composites were enhanced as a result of synthetic fiber’s incorporation. Baihaqi et al. [131] revealed the effects of fiber content and hybridization on bending and torsional strengths of hybrid epoxy composites reinforced with carbon and sugar palm fibers. Improvements of the flexural and torsion properties of nonhybrid composites at 15 wt.% fiber loading were found to be 7.40% and 75.61%, respectively, over the composites with 5, 10, and 20 wt.% fiber loading. The findings from this study suggested that the hybrid composites exhibited better flexural and torsion properties performance. The findings on the mechanical properties of hybrid synthetic/natural fiber-reinforced thermosets composites stated in previous studies are shown in Table 3. Overall, the mechanical characteristics of the studied composites were found to improve as a result of synthetic fiber’s incorporation. Khanam et al. [132] investigated the properties of flexural, tensile, and chemical resistance of sisal/carbon fiber-reinforced polyester hybrid composites. The hybrid composites’ tensile and flexural strengths and tensile and flexural moduli were observed to rise with the carbon fiber content increment. Ramesh et al. [133] carried out a mechanical properties investigation of the composite of glass/sisal/jute hybrid polyester. It was noted that the additions of glass fiber with jute, as well as the sisal fiber-reinforced composite, had resulted in improvements in the mechanical properties. Among all composites, the glass/jute composite exhibited maximum tensile strength, glass/jute/sisal possessed maximum flexural load, and glass/sisal showed maximum impact strength.

Kumar et al. [134] presented the positive effect of hybridization, in terms of mechanical properties, due to the incorporation of glass and hybrid banana fibers into polypropylene matrix. The maximum tensile, impact, and flexural strengths of the hybrid composite were 24.59 MPa, 29.37 J/m, and 227.81 MPa, correspondingly, which were 3, 53, and 19, correspondingly, more than the polypropylene matrix, alone. Al Maadeed et al. [135] worked on date palm wood flour/glass fiber-reinforced hybrid polypropylene composites and observed increments in tensile strength and modulus of the composite resulting from glass fibers reinforcement. Adding only 5% glass fiber had found to increase the composite’s tensile strength by 18%.

A number of researchers worked on the effect of natural fiber addition on the mechanical properties of natural fiber-reinforced thermosets polymer composites. The mechanical properties of hybrid natural/natural fiber-reinforced thermosets composites from literature are displayed in Table 4. The findings concluded that the mechanical properties of the composites were increased after natural fiber incorporation, having comparatively long elongation. Shanmugam and Thiruchitrambalam [136] studied the mechanical characteristics of unidirectional palm stalk fiber/jute fiber-reinforced polyester matrix composite and revealed that the addition of jute fiber into the composite had enhanced the studied mechanical properties. The P50J50 hybrid composite showed 11 and 28% improvements in tensile and flexural strengths, respectively, over the palm polyester composite. Srinivasan et al. [137] reported findings on the mechanical characteristics of banana epoxy and hybrid flax composites fabricated via the hand lay-up technique. The hybrid composites possessed more excellent flexural and impact properties than the glass-reinforced epoxy composite.

The summary of various findings on the mechanical properties of hybrid natural/natural fiber-reinforced thermoplastic composites from previous works is shown in Table 4. The mechanical properties of natural fiber-reinforced thermoplastic composites were improved as a result of the incorporation of natural fiber with relatively high strain. Asaithambi et al. [138,139] studied the mechanical properties of hybrid banana/sisal-reinforced polylactic acid composite. Compared to banana-reinforced Poly(lactic acid) (PLA) and neat PLA, improved mechanical properties were observed in the composites incorporated with high-strength sisal fiber with banana fiber–PLA composite. The hybrid composites exhibited enhanced tensile strength and modulus by 21 and 40%, respectively, compared to neat PLA composites, as well as improvements in flexural strength and modulus by 12 and 45%, respectively, compared to neat PLA composites. Khan et al. [140] reported findings of the hybrid composites of jute and cellulose (cordenka) with polypropylene, fabricated via the injection molding method. The hybrid composites with 75 wt.% cordenka and 25 wt.% jute exhibited better mechanical characteristics, compared to other natural/natural hybrid composites, as shown in Table 4.

### 4.2. Impact Properties

The resistance provided by a hybrid composite against impact load without failure is determined by impact strength. Therefore, before its implementation in any structural applications, it is essential for a design engineer or scientist to know the impact properties of the component. The hybrid polymer composite analysis consisting of kenaf bast fiber and fiberglass/kevlar as reinforcements in the matrix of polyester resin explores the impact properties of composites [175]. In this study, different percentages of volume fraction of kenaf fiber to hybrid fiberglass/kevlar polymeric composite were used (0%, 15%, 45%, 60%, and 75%) based on the volume fraction. The results of energy absorption and impact strength of kenaf fiber-reinforced fiberglass/kevlar hybrid polymeric composites were found to increase, due to the increased kenaf fiber contents at 15, 45, and 60 vol %, and decrease at 75%, due to manufacturing defects that can be found in the sample, i.e., interfacial adhesion, voids, and fiber pull-outs. In addition, kenaf fiber-reinforced fiberglass/kevlar hybrid polymeric composite showed the highest value of energy absorbed and impacts strength at 8.71 J and 0.085 J/mm^2^, respectively. It can be concluded that the manufacturing defects had influenced the performance of impact behaviors of kenaf fiber-reinforced fiberglass/kevlar hybrid polymer composite.

Further research on the impact strength of neem, abaca, and glass fibers hybrid composites as reinforcements in epoxy polymer matrices was performed by Kaliappan [176]. The various composite specimens were produced in laminates with various fiber orientations, such as horizontal, vertical, and 45° inclined. The hybrid neem composite was found to be one of the reinforcements with a 45° fiber orientation that held better impact strength than the other orientations. However, more cavities and fiber cracks were found in the specimen, leading to a decline in material strength. This can be stopped by the correct distribution of the mixture of resin hardeners. In addition, researchers had proposed that abaca/glass hybrid epoxy composite had greater impact strength than jute/glass and composites of abaca/jute/glass [143]. This improved abaca composite performance was due to improved adhesive strength with epoxy. Other than that, a study on fracture behavior of all-cellulose composite and acc laminates during impact loading by Huber et al. [177] found the matrix failures, such as matrix phase cracking parallel to the fibers; laminate layer delamination, due to interlaminar stresses; fiber failure, such as breakage and buckling; and complete laminate penetration [178]. In addition, all the samples tested for puncture impact showed a pyramidal-type failure that suggested the presence of a rigid matrix. Compliant matrices appeared to demonstrate major deformation, a bulged surface, and fiber pull-outs [177]. From this study, at the fracture surface, there was no evidence of delamination of laminae, suggesting strong interlaminar adhesion within the acc laminate. The preferred damage mode in these acc laminates tended to be broad intralaminar delamination involving the splitting of the matrix process connecting individual fibers and fiber bundles within the laminae.

### 4.3. Tensile Properties

In a study to determine the mechanical properties of the intralayer abaca–jute–glass fiber-reinforced composite, Ramnath et al. [123] studied the tensile properties of composites. There were five layers of each composite, surrounded by two layers of triple-layered jute, abaca, and glass fibers [123]. The second layer fibers in category I were orthogonal to the first and third layer fibers. Both fibers were parallel to each other in cctegories II and III, and the fibers in the second layer were at a 45° angle to the first and third layers. Based on the tensile test, sample 3, with a higher volume of abaca content than jute, was far superior to the other two samples. In tensile testing, samples provided with a 45° fiber orientation (category III) excelled, followed by category II and category I orientation, with regard to the orientation of the fibers. This means that, as the abaca fiber composition increased, the tensile properties of the composites also improved. The researchers reported that sample 3 showed better properties, compared to other samples. This suggests that composites with higher abaca fiber composition have superior delamination properties. The samples with higher abaca content demonstrated stronger tensile properties in the tensile test. The poor bonding between fiber and resin and the presence of voids created by the method of hand lay-up, however, might reduce the strength of the composite.

**Figure 7:**
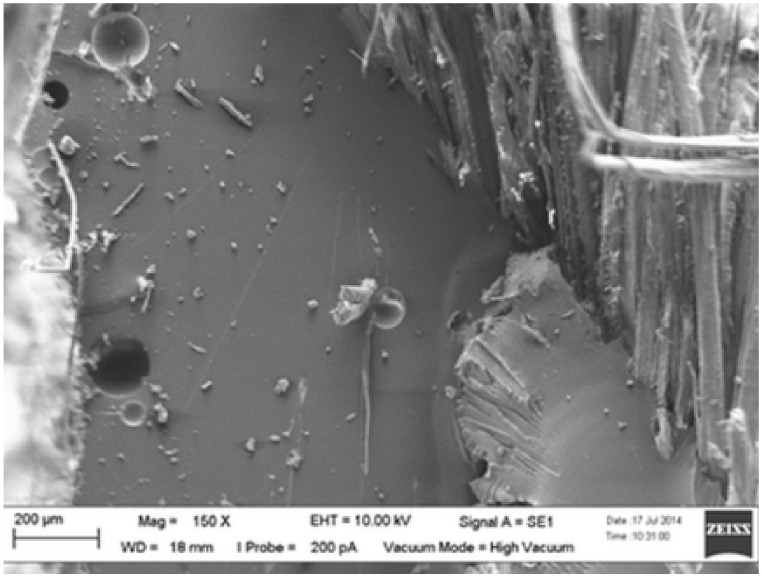
Scanning electron microscope images of tensile fracture sample of hybrid with layering sequences: k/a/k. Reproduced with copyright permission from Yahaya et al. [150].

**Figure 8:**
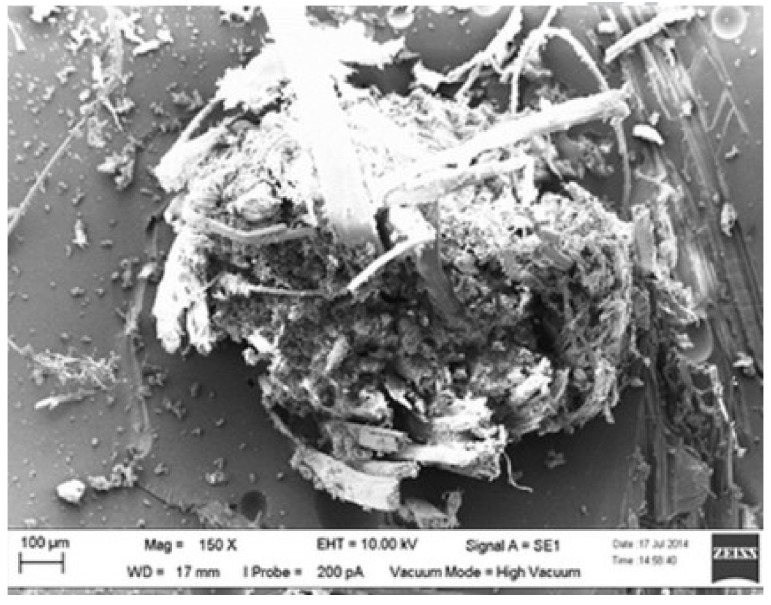
Scanning electron microscope images of tensile fracture sample of hybrid with layering sequences: k/a/k. Reproduced with copyright permission from Yahaya et al. [150].

Another study was performed on the impact of layering and chemical treatment sequences on the tensile properties of woven kenaf–kevlar composites by Yahaya et al. [150]. The two different samples were fabricated with different layering sequences with a: kevlar and k: kenaf (treated with NaOH), which were k/a/k and a/k/a/k. Average tensile strength values of 99.4 and 123 MPa, respectively, were demonstrated by three-layer and four-layer hybrid laminates, depending on the outcome. The increase in four-layer hybrid composite tensile strength was due to the addition of one more layer of kevlar, and that was also proven to improve the tensile strength of the handled kenaf/epoxy (k/et), compared to the untreated kenaf/epoxy (k/e). According to Ibrahim, by eliminating natural and artificial impurities, the alkaline treatment improved the characteristics of fiber surface adhesion, thus improving the interaction of the fiber–matrix by removing lignin and hemicellulose, resulting in greater fiber integration with the matrix [179]. Compared with the a/k/a sample, the fiber pull-outs, fiber–matrix incompatibility, and matrix cracking and voids in the k/a/k sample were observed, as shown in Figure 7 and Figure 8.

An analysis was carried out by Aslan et al. [180] on sisal and glass fiber-reinforced polypropylene composites with 42 wt.% of total fiber content. The tensile properties of hybrid composites were investigated, showing that sisal/glass composite showed a lower tensile module value than sisal/carbon composite, with increments in the contents of carbon and glass fibers in sisal/carbon and sisal/glass hybrid composite, respectively [180]. For both composites, the tensile modulus values were also increased, which caused the tensile strength of the hybrid composite to increase. In addition, the scanning electron microscopy analysis showed that sisal/carbon and sisal/glass fibers with polypropylene matrix had low interfacial adhesion. In hybrid composites, small and wide cavities and holes can be found, due to debonding and pulling out separate long fibers on fracture surfaces. However, due to the smaller pull-out fibers and smaller gaps than larger cavities, sisal/glass hybrid composite displayed greater fiber penetration, smoother matrix surfaces, and longer fiber pull-outs of sisal/carbon hybrid composite surfaces.

## 5. Conclusions

In the manufacturing of boat structures, natural fibers are seen as possible substitutes. Although natural fibers possess the advantages of being low-cost, eco-friendly, and low-density, they are not free from problems. A serious problem of natural fiber hybrid composite encountered, especially during the manufacturing process, is delamination, and that has become the most common type of composite material failure. Due to high interlaminar stress associated with the normal very low through-thickness intensity, this phenomenon can occur. However, the material failure is not only affected by delamination of the composites, but it is also influenced by manufacturing defects during the process of manufacturing. Usually, it is possible to find the defects caused by a production process, such as gap, resin-rich zone, undispersed crosslinker pocket, misaligned fibers, and areas where resin improperly wets fiber. All these flaws contributed to the poor performance of the fiber-reinforced hybrid composite’s composite strength and mechanical properties.

However, based on this review, there are some recommendations to control the delamination and manufacturing defects in the hybrid composites.

- By heating the reinforcement in an oven to 565°C for several hours, the creation of voids can be managed. Compared with the initial reinforcement, the void content then decreased dramatically. This is to modify the surface energy of the fibers by removing the treatment of the surface. In addition, during the fabrication of the specimen, the surrounding temperature must be at constant room temperature to avoid the air from trapping in the specimen and use a roller to remove the trapped air bubbles for hand lay-up technique.
- In order to circumvent the region where resin has poorly wetted the fiber and in order to alter the properties of natural fibers, the fibers must undergo chemical treatments using silane, acetic acid, acrylic acid, etc. This is to promote adhesion by chemically coupling the adhesive to the substrate by enhancing the strength of the fiber fitness and the strengthened natural fiber matrix adhesion.
- Additionally, areas of resin-rich zones in the composite can be controlled by using digital image processing techniques, where the machine can monitor the fiber and matrix distribution in the composite to determine the radiometric properties by plotting the intensity component of the image as a depth map.
- Fiber pull-outs usually occur due to the resin that is not well-distributed through the fiber’s surface and will affect the interfacial between the fiber and matrix. It is suggested that the resin and the optimum fiber contents used must be spread well in the composite during the fabrication.
- The phenomenon of fibers’ misalignment in the composite material can be controlled by (1) keeping the laminate thickness below certain limits to minimize exothermal heat generation; (2) using low forming speeds by generating lower resistance to interply and intraply shearing, thus allowing the blank to deform more easily; (3) preconsolidation every four to five layers using a vacuum bag; and (4) using rubber pads for better pressure distribution.

## Figures and Tables

**Table 1:** Chemical composition of selected common natural fibers.

Fibers	Holocellulose (wt.%)						
	Cellulose (wt.%)	Hemicellulose (wt.%)	Lignin (wt.%)	Ash (wt.%)	Extractives (wt.%)	Crystallinity (%)	Ref.
Sugar palm	43.88	7.24	33.24	1.01	2.73	55.8	[69]
Wheat straw	43.2 ± 0.15	34.1 ± 1.2	22.0 ± 3.1	-	-	57.5	[87]
Soy hull	56.4 ± 0.92	12.5 ± 0.72	18.0 ± 2.5	-	-	59.8	[87]
Arecanut husk	34.18	20.83	31.60	2.34	-	37	[88]
Helicteres isora plant	71 ± 2.6	3.1 ± 0.5	21 ± 0.9	-	-	38	[89]
Pineapple leaf	81.27 ± 2.45	12.31 ± 1.35	3.46 ± 0.58	-	-	35.97	[90]
Ramie	69.83	9.63	3.98	-	-	55.48	[91]
Oil palm mesocarp fiber (OPMF)	28.2 ± 0.8	32.7 ± 4.8	32.4 ± 4.0	-	6.5 ± 0.1	34.3	[92]
Oil palm empty fruit bunch (OPEFB)	37.1 ± 4.4	39.9 ± 0.75	18.6 ± 1.3	-	3.1 ± 3.4	45.0	[92]
Oil palm frond (OPF)	45.0 ± 0.6	32.0 ± 1.4	16.9 ± 0.4	-	2.3 ± 1.0	54.5	[92]
Oil palm empty fruit bunch (OPEFB) fiber	40 ± 2	23 ± 2	21 ± 1	-	2.0 ± 0.2	40	[93]
Rubber wood	45 ± 3	20 ± 2	29 ± 2	-	2.5 ± 0.5	46	[93]
Curauna	70.2 ± 0.7	18.3 ± 0.8	9.3 ± 0.9	-	-	64	[94]
Banana	7.5	74.9	7.9	0.01	9.6	15.0	[95]
Sugarcane bagasse	43.6	27.7	27.7	-	-	76	[96]
Kenaf bast	63.5 ± 0.5	17.6 ± 1.4	12.7 ± 1.5	2.2 ± 0.8	4.0 ± 1.0	48.2	[97]
Phoenix dactylifera palm leaflet	33.5	26.0	27.0	6.5	-	50	[98]
Phoenix dactylifera palm rachis	44.0	28.0	14.0	2.5	-	55	[98]
Kenaf core powder	80.26		23.58	-	-	48.1	[99]
Water hyacinth	42.8	20.6	4.1	-	-	59.56	[100]
Wheat straw	43.2 ± 0.15	34.1 ± 1.2	22.0 ± 3.1	-	-	57.5	[101]
Sugar beet	44.95 ± 0.09	25.40 ± 2.06	11.23 ± 1.66	17.67 ± 1.54	-	35.67	[102]
Mengkuang leaves	37.3 ± 0.6	34.4 ± 0.2	24 ± 0.8		2.5 ± 0.02	55.1	[103]

**Table 2:** Physical and mechanical properties of natural and synthetic fibers.

Fibers	Diameter (µm)	Density (g/cc)	Young’s Modulus (GPa)	Tensile Strength (MPa)	References
Jute	25–250	1.3–1.49	13–26.5	393–800	[123]
Sisal	100–300	1.44	9–20	227–400	[127,128]
Abaca	10–30	1.5	31.1–33.6	430–813	[123]
Pineapple	20–80	1.44	34.5–82.5	413–1627	[127]
Bamboo	-	0.91	35.91	503	[127]
Kenaf	-	1.45	53	930	[129]
Banana	-	1.35	3.5	56	[130]
Coconut	-	-	3–5	140–225	[130]
Ramie	20–80	1–1.55	24.5–128	400–1000	[127]
Glass	15–25	2.55	70–73	2000–3500	[123]
Kevlar	11.9	1.4	124	300	[127]

**Table 3:** Mechanical properties of synthetic/natural fiber-reinforced hybrid polymer composites.

Matrix	Fibers	Methods of Manufacturing	FS (MPa)	FM (GPa)	TS (MPa)	TM (GPa)	Impact Strength/Energy	Ref.
Thermoset								
Unsaturated Polyester	Carbon/sugar palm	Hand lay-up	87	3.3	-	-	-	[131]
Epoxy	Carbon/flax	Compression molding	318.83	28.83	126.3	2.9	-	[141]
Polyester	Carbon/sisal	Hand lay-up	131.48	7.97	38.3	1.97	-	[132]
Epoxy	Carbon/unidirectional (UD) cellulosic flax	Compression molding	318.83	28.83	126.3	2.9	-	[141]
Polyester	Glass fabric/woven jute weave	Hand lay-up	12.38	159.85	125	12.5	-	[142]
Epoxy	Glass/abaca	Hand lay-up	12.5	1.38	44.5	0.27	16 J	[143]
Epoxy	Glass/abaca/jute	Hand lay-up	12.1	1.452	57	0.29	12 J	[143]
Polyester	Glass/curaua	Hot compression molding	170–180	9.5–10.5	90–93	0.0095–0.01	-	[144]
Phenolic	Glass/flax	Compression molding	-	-	39.7	39.7	-	[145]
Polyester	Glass/jute	Pultrusion process	343.32	24.6	266.22	27.5	-	[146]
Polyester	Glass/jute		-	-	229.54	-	10 J	[133]
Epoxy	Glass/jute	Hand lay-up	11.9	1.216	46.5	0.25	15 J	[143]
Polyester	Glass/kenaf	-	453.22	3	38–42	2–3	-	[147]
Polyester	Glass/sisal	Hand lay-up	89.2	-	65.2	-	-	[148]
Epoxy	Glass/sisal/banana	Compression molding	163	-	104	2.35	12.8 J	[149]
Polyester	Glass/sisal/jute	Hand lay-up	-	-	200	-	12 J	[133]
Polyester	Glass/sisal/red mud	Hand lay-up	98.1	-	45.2	5.95	-	[148]
Epoxy	Aramid/kenaf	Hand lay-up	45.7	1.96	64.7	5.29	50.1 kJ/m^2^	[150]
Epoxy	Aramid/kenaf	Hand lay-up	-	-	-	-	324.4 J	[151]
Epoxy	Carbon/cross-ply (CP) cellulosic flax	Compression molding	145	9.71	284.8	11.9	-	[141]
Unsaturated polyester	Carbon/sisal	Hand lay-up	131.48	7.97	38.3	1.97	-	[152]
Epoxy	Glass/banana/flax	Hand lay-up	9.76		39		12 J	[137]
Epoxy	Glass/basalt	Hand lay-up	-	-	210.3	14.1	-	[153]
Epoxy	Glass/flax/basalt	Vacuum infusion	137.95	8.02	153.16	8.11	-	[154]
Epoxy	Glass/hem/basalt	Vacuum infusion	126.22	5.9	128.84	6.64	-	[154]
Polyester	Glass/sisal	Hand lay-up	-	-	176.2	-	18 J	[133]
Polyester	Glass/sisal	Hand lay-up	-	-	-	-	238 kJ/m^2^	[155]
Phenolic	Glass/unidirectional flax	Compression molding		-	412.5	40.8	-	[145]
Epoxy	Kevlar/kenaf	Hand lay-up	15	1.3	202	3.4	34.86 J	[156]
Epoxy	Kevlar/kenaf	Hand lay-up	105	3.26	164.6	4.39	-	[157]
Epoxy	Nylon fabric/coir pith	Compression molding	106.52	-	11.3	-	359 J/m	[158]
Novolac phenolic (PF)	Woven glass/montmorilonite	Compression molding	313	14.4	250	11.3	-	[159]
**Thermoplastic**								
Polypropylene	Cordenka/jute	Pultrusion technique	-	-	72	3.2	79 kJ/m^2^	[140]
Polypropylene	Cordenka/soft wood	Injection Molding	-	122	86	8.5	-	[160]
Polypropylene	E-glass /date palm	Injection molding	-	-	20.5	12.25	-	[161]
Polypropylene	Glass/ banana	Injection molding	270.86	0.794	24.59	0.322	29.39 J/m	[134]
Polypropylene	Glass/flax	Injection Molding	65–66	4.5–4.6	38–39	2.1–2.15	-	[162]
Polypropylene	Glass/hemp	-	366	11.3	-	-	-	[163]

**Table 4:** Mechanical properties of natural fiber/natural fiber-reinforced hybrid polymer composites.

Matrix	Fibers	Methods of Manufacturing	FS (MPa)	FM (GPa)	TS (MPa)	TM (GPa)	Impact Strength/Energy	Ref.
Thermoset								
Epoxy	Abaca/jute/glass	Hand lay-up	3.169	-	45.63	0.228	4.66 J	[123]
Epoxy	Flax/banana	Hand lay-up	13.54	-	30	-	16 J	[137]
Epoxy	Flax/hemp	Compression molding	-	-	40–60	-	14–20 kJ/m^2^	[164]
Epoxy	Flax/hemp/basalt	Vacuum infusion	128.46	7.45	115.97	7.69	-	[154]
Epoxy	Jute/banana	Hand-lay-up	59.84	9.17	18.96	0.724	18.23 kJ/m^2^	[126]
Epoxy	Jute/banana	Hand lay-up	59.84	9.17	18.96	0.724	18.23 kJ/m^2^	[126]
Epoxy	Jute/oil palm	Hand lay-up	49	3.07	-	-	57.0 J/m	[165]
Epoxy	Jute/oil palm	Hand lay-up	-	-	25.3	2.62	-	[166]
Epoxy	Jute/oil palm	Hand lay-up	-	-	37.9	3.31	-	[166]
Epoxy	Jute/oil palm	Hand lay-up	49	3.07	-	-	57.0 J/m	[165]
Polyester	Jute/palm leaf stalk	Compression molding	164	18.23	83.3	3.78	26.02 kJ/m^2^	[136]
Epoxy	Jute/wool	Hand lay-up	72.7	5.65	40.24	3.5	9 J	[167]
Polyester	Kenaf/banana	Hand lay-up	172.2	-	110	-	23 kJ/m^2^	[168]
Polyester	Palm leaf/jute	Compression molding	145.66	17.95	64.3	2.45	27.01 kJ/m^2^	[136]
Epoxy	Sisal/banana	Hand lay-up	59.687	9.13	18.66	0.682	17.9 kJ/m^2^	[169]
Polyester	Sisal/roselle	Hand lay-up	76.5	-	58.7	-	1.32 kJ/m^2^	[170]
Polyester	Sisal/roselle	Compression molding	51.3	-	32.4	-	1.41 kJ/m^2^	[171]
Epoxy	Wool and jute	Hand lay-up	76.01	6.1	50.51	4.97		[167]
Epoxy	Woven jute/banana	Hand lay-up	91.66	9.44	54.76	13.69	73.33 J/m	[172]
**Thermoplastic**								
High density polyethylene	Agave/pine	Extrusion and injection molding	28.5	1.173	24	0.62	53 J/m	[173]
Polypropylene	Cordenka/jute	Injection molding	-	-	72	3.2	79 kJ/m^2^	[140]
Acrylate	Hemp/kenaf	Compression molding	-	-	11.3	1.2–3	6–40 kJ/m^2^	[174]
Poly(lactic acid) (PLA)	Hemp/kenaf	Compression molding	-	-	61	7.763	11.8 kJ/m^2^	[174]
Poly(lactic acid) (PLA)	Hemp/lyocell	Compression molding	-	-	71.5	7.034	24.7 kJ/m^2^	[174]
Bisphenol-C-formaldehyde	Jute/cane sugar husk	Hand lay-up	48	-	12	-	-	[162]
Bisphenol-C-formaldehyde	Jute/jamun flower husk	Hand lay-up	41	-	12	-	-	[162]
Bisphenol-C-formaldehyde	Jute/rice husk	Hand lay-up	14	-	10	-	-	[162]
Bisphenol-C-formaldehyde	Jute/wheat husk	Hand lay-up	29	-	17	-	-	[162]
Poly lactic acid	Sisal/banana	Injection molding	91	4.2	57	1.7	31.5 kJ/m^2^	[138]

FS = flexural strength; FM = flexural strength; TS = tensile strength; TM = tensile modulus; GPa = Giga Pascals; MPa = Mega Pascals.

## Data Availability

The data presented in this study are available on request from the corresponding author.

## References

[1] EL-Wazery M.S., EL-Elamy M.I., Zoalfakar S.H. (2017). Mechanical properties of glass fiber reinforced polyester composites. Int. J. Appl. Sci. Eng..

[2] Shalwan A., Yousif B. (2013). In state of art: Mechanical and tribological behaviour of polymeric composites based on natural fibres. Mater. Des..

[3] Mohammed L., Ansari M.N.M., Pua G., Jawaid M., Islam M.S. (2015). A Review on Natural Fiber Reinforced Polymer Composite and Its Applications. Int. J. Polym. Sci..

[4] Nurazzi N.M., Khalina A., Sapuan S.M., Ilyas R.A., Rafiqah S.A., Hanafee Z.M. (2020). Thermal properties of treated sugar palm yarn/glass fiber reinforced unsaturated polyester hybrid composites. J. Mater. Res. Technol..

[5] Aisyah H.A., Paridah M.T., Sapuan S.M., Khalina A., Berkalp O.B., Lee S.H., Lee C.H., Nurazzi N.M., Ramli N., Wahab M.S. (2019). Thermal Properties of Woven Kenaf/Carbon Fibre-Reinforced Epoxy Hybrid Composite Panels. Int. J. Polym. Sci..

[6] Sapuan S.M., Aulia H.S., Ilyas R.A., Atiqah A., Dele-Afolabi T.T., Nurazzi M.N., Supian A.B.M., Atikah M.S.N. (2020). Mechanical properties of longitudinal basalt/woven-glass-fiber-reinforced unsaturated polyester-resin hybrid composites. Polymers.

[7] Kumar T.S.M., Chandrasekar M., Senthilkumar K., Ilyas R.A., Sapuan S.M., Hariram N., Rajulu A.V., Rajini N., Siengchin S. (2020). Characterization, Thermal and Antimicrobial Properties of Hybrid Cellulose Nanocomposite Films with in-Situ Generated Copper Nanoparticles in Tamarindus indica Nut Powder. J. Polym. Environ..

[8] Atiqah A., Jawaid M., Sapuan S.M., Ishak M.R., Ansari M.N.M., Ilyas R.A. (2019). Physical and thermal properties of treated sugar palm/glass fibre reinforced thermoplastic polyurethane hybrid composites. J. Mater. Res. Technol..

[9] Norizan M.N., Abdan K., Ilyas R.A. (2019). Effect of water absorption on treated sugar palm yarn fibre/glass fibre hybrid composites. Proceedings of the Prosiding Seminar Enau Kebangsaan.

[10] Ibrahim M.I.J., Sapuan S.M., Zainudin E.S., Zuhri M.Y.M. (2020). Preparation and characterization of cornhusk/sugar palm fiber reinforced Cornstarch-based hybrid composites. J. Mater. Res. Technol..

[11] Afzaluddin A., Jawaid M., Salit M.S., Ishak M.R. (2019). Physical and mechanical properties of sugar palm/glass fiber reinforced thermoplastic polyurethane hybrid composites. J. Mater. Res. Technol..

[12] Alsubari S., Zuhri M.Y.M., Sapuan S.M., Ishak M.R., Ilyas R.A., Asyraf M.R.M. (2021). Potential of Natural Fiber Reinforced Polymer Composites in Sandwich Structures: A Review on Its Mechanical Properties. Polymers.

[13] Ramasamy M., Arul Daniel A., Nithya M., Sathees Kumar S., Pugazhenthi R. (2020). Characterization of natural—Synthetic fiber reinforced epoxy based composite—Hybridization of kenaf fiber and kevlar fiber. Mater. Today Proc..

[14] Asumani O.M.L., Reid R.G., Paskaramoorthy R. (2012). The effects of alkali-silane treatment on the tensile and flexural properties of short fibre non-woven kenaf reinforced polypropylene composites. Compos. Part A Appl. Sci. Manuf..

[15] Mohd Nurazzi N., Asyraf M.R.M., Khalina A., Abdullah N., Sabaruddin F.A., Kamarudin S.H., Ahmad S., Mahat A.M., Lee C.L., Aisyah H.A. (2021). Fabrication, Functionalization, and Application of Carbon Nanotube-Reinforced Polymer Composite: An Overview. Polymers.

[16] Chermoshentseva A.S., Pokrovskiy A.M., Bokhoeva L.A. (2016). The behavior of delaminations in composite materials—Experimental results. IOP Conf. Ser. Mater. Sci. Eng..

[17] Wisnom M.R. (2012). The role of delamination in failure of fibre-reinforced composites. Philos. Trans. R. Soc. A Math. Phys. Eng. Sci..

[18] Imran M., Khan R., Badshah S. (2018). A review on the effect of delamination on the performance of composite plate. Pak. J. Sci. Ind. Res. Ser. A Phys. Sci..

[19] Hwang S.-F., Mao C.-P. (2001). Failure of delaminated interply hybrid composite plates under compression. Compos. Sci. Technol..

[20] Potter K., Khan B., Wisnom M., Bell T., Stevens J. (2008). Variability, fibre waviness and misalignment in the determination of the properties of composite materials and structures. Compos. Part A Appl. Sci. Manuf..

[21] Ghayoor H., Marsden C.C., Hoa S.V., Melro A.R. (2019). Numerical analysis of resin-rich areas and their effects on failure initiation of composites. Compos. Part A Appl. Sci. Manuf..

[22] Rajak D.K., Pagar D.D., Menezes P.L., Linul E. (2019). Fiber-reinforced polymer composites: Manufacturing, properties, and applications. Polymers.

[23] Suriani M.J., Ali A., Khalina A., Sapuan S.M., Abdullah S. (2012). Detection of Defects in Kenaf/Epoxy using Infrared Thermal Imaging Technique. Procedia Chem..

[24] Elkington M., Bloom D., Ward C., Chatzimichali A., Potter K. (2015). Hand layup: Understanding the manual process. Adv. Manuf. Polym. Compos. Sci..

[25] Talreja R. (2015). Manufacturing defects in composites and their effects on performance. Polym. Compos. Aerosp. Ind..

[26] Mehdikhani M., Gorbatikh L., Verpoest I., Lomov S.V. (2019). Voids in fiber-reinforced polymer composites: A review on their formation, characteristics, and effects on mechanical performance. J. Compos. Mater..

[27] Ornaghi H.L., Neves R.M., Monticeli F.M., Almeida J.H.S. (2020). Viscoelastic characteristics of carbon fiber-reinforced epoxy filament wound laminates. Compos. Commun..

[28] Liu X., Chen F. (2016). A review of void formation and its effects on the mechanical performance of carbon fiber reinforced plastic. Eng. Trans..

[29] Lundström T.S., Gebart B.R., Lundemo C.Y. (1993). Void Formation in RTM. J. Reinf. Plast. Compos..

[30] Lundström T.S., Gebart B.R. (1994). Influence from process parameters on void formation in resin transfer molding. Polym. Compos..

[31] Afendi M., Banks W.M., Kirkwood D. (2005). Bubble free resin for infusion process. Compos. Part A Appl. Sci. Manuf..

[32] Kang M.K., Lee W., Hahn H.T. (2000). Formation of microvoids during resin-transfer molding process. Compos. Sci. Technol..

[33] Dong C., Tsai T.C. (2010). Formation of resin-rich zones in composites processing. Adv. Mater. Res..

[34] Glinz J., Šleichrt J., Kytýř D., Ayalur-Karunakaran S., Zabler S., Kastner J., Senck S. (2021). Phase-contrast and dark-field imaging for the inspection of resin-rich areas and fiber orientation in non-crimp vacuum infusion carbon-fiber-reinforced polymers. J. Mater. Sci..

[35] Koutsonas S. (2018). Modelling race-tracking variability of resin rich zones on 90° composite 2.2 twill fibre curved plate. Compos. Sci. Technol..

[36] Haesch A., Clarkson T., Ivens J., Lomov S.V., Verpoest I., Gorbatikh L. (2015). Localization of carbon nanotubes in resin rich zones of a woven composite linked to the dispersion state. Nanocomposites.

[37] Holmberg J.A., Berglund L.A. (1997). Manufacturing and performance of RTM U-beams. Compos. Part A Appl. Sci. Manuf..

[38] Ahmadian H., Yang M., Soghrati S. (2020). Effect of resin-rich zones on the failure response of carbon fiber reinforced polymers. Int. J. Solids Struct..

[39] Idrees M., Ibrahim A.M.H., Tekerek E., Kontsos A., Palmese G.R., Alvarez N.J. (2021). The effect of resin-rich layers on mechanical properties of 3D printed woven fiber-reinforced composites. Compos. Part A Appl. Sci. Manuf..

[40] Placet V. (2009). Composites: Part A Characterization of the thermo-mechanical behaviour of Hemp fibres intended for the manufacturing of high performance composites. Compos. Part A.

[41] Placet V., Cisse O. (2012). Influence of environmental relative humidity on the tensile and rotational behaviour of hemp fibres. J. Mater. Sci..

[42] Cook W.D., Mehrabi M., Edward G.H. (1999). Ageing and yielding in model epoxy thermosets. Polymer.

[43] Kumar D.S., Shukla M.J., Mahato K.K., Rathore D.K., Prusty R.K., Ray B.C. (2015). Effect of post-curing on thermal and mechanical behavior of GFRP composites. IOP Conf. Ser. Mater. Sci. Eng..

[44] Symp M. (2005). © 2005 WILEY-VCH Verlag GmbH & KGaA, Weinheim. Angew. Chem. Int. Ed..

[45] Masseteau B., Michaud F., Irle M., Roy A., Alise G. (2014). Composites: Part A An evaluation of the effects of moisture content on the modulus of elasticity of a unidirectional flax fiber composite. Compos. Part A.

[46] Gomina M. (2014). Effects of the hygrothermal environment on the mechanical properties of flax fibres. J. Compos. Mater.

[47] Thor M., Sause M.G.R., Hinterhölzl R.M. (2020). Mechanisms of Origin and Classification of Out-of-Plane Fiber Waviness in Composite Materials—A Review. J. Compos. Sci..

[48] Hsiao H.M., Daniel I.M. (1996). Effect of fiber waviness on stiffness and strength reduction of unidirectional composites under compressive loading. Compos. Sci. Technol..

[49] Parlevliet P.P., Bersee H.E.N., Beukers A. (2007). Residual stresses in thermoplastic composites—a study of the literature. Part III: Effects of thermal residual stresses. Compos. Part A Appl. Sci. Manuf..

[50] Baran I., Cinar K., Ersoy N., Akkerman R., Hattel J.H. (2017). A Review on the Mechanical Modeling of Composite Manufacturing Processes. Arch. Comput. Methods Eng..

[51] Soll W., Gutowski T.G. Forming thermoplastic composite parts. Proceedings of the 33rd International SAMPE Symposium and Exhibition.

[52] Mallick P.K., Mallick P.K. (2018). Processing of Polymer Matrix Composites.

[53] Aström B.T., Bhattacharyya D. (1997). Thermoplastic composite sheet forming: Materials and manufacturing techniques. Composite Materials Series.

[54] Hubert P., Centea T., Grunefelder L., Nutt S., Kratz J., Levy A. (2018). Out-of-Autoclave Prepreg Processing.

[55] Hassan M.H., Othman A.R., Kamaruddin S. (2017). A review on the manufacturing defects of complex-shaped laminate in aircraft composite structures. Int. J. Adv. Manuf. Technol..

[56] Ilyas R.A., Sapuan S.M. (2020). Biopolymers and Biocomposites: Chemistry and Technology. Curr. Anal. Chem..

[57] Ilyas R.A., Sapuan S.M. (2020). The Preparation Methods and Processing of Natural Fibre Bio-polymer Composites. Curr. Org. Synth..

[58] Li X., Tabil L.G., Panigrahi S. (2007). Chemical treatments of natural fiber for use in natural fiber-reinforced composites: A review. J. Polym. Environ..

[59] Atikah M.S.N., Ilyas R.A., Sapuan S.M., Ishak M.R., Zainudin E.S., Ibrahim R., Atiqah A., Ansari M.N.M., Jumaidin R. (2019). Degradation and physical properties of sugar palm starch/sugar palm nanofibrillated cellulose bionanocomposite. Polimery.

[60] Abral H., Ariksa J., Mahardika M., Handayani D., Aminah I., Sandrawati N., Sapuan S.M., Ilyas R.A. (2019). Highly transparent and antimicrobial PVA based bionanocomposites reinforced by ginger nanofiber. Polym. Test..

[61] Syafri E., Sudirman, Mashadi, Yulianti E., Deswita, Asrofi M., Abral H., Sapuan S.M., Ilyas R.A., Fudholi A. (2019). Effect of sonication time on the thermal stability, moisture absorption, and biodegradation of water hyacinth (Eichhornia crassipes) nanocellulose-filled bengkuang (Pachyrhizus erosus) starch biocomposites. J. Mater. Res. Technol..

[62] Mukaffa H., Asrofi M., Sujito, Asnawi, Hermawan Y., Sumarji, Qoryah R.D.H., Sapuan S.M., Ilyas R.A., Atiqah A. (2021). Effect of alkali treatment of piper betle fiber on tensile properties as biocomposite based polylactic acid: Solvent cast-film method. Mater. Today Proc..

[63] Ilyas R.A., Sapuan S.M., Atikah M.S.N., Asyraf M.R.M., Rafiqah S.A., Aisyah H.A., Nurazzi N.M., Norrrahim M.N.F. (2021). Effect of hydrolysis time on the morphological, physical, chemical, and thermal behavior of sugar palm nanocrystalline cellulose (Arenga pinnata (Wurmb.) Merr). Text. Res. J..

[64] Rozilah A., Jaafar C.N.A., Sapuan S.M., Zainol I., Ilyas R.A. (2020). The Effects of Silver Nanoparticles Compositions on the Mechanical, Physiochemical, Antibacterial, and Morphology Properties of Sugar Palm Starch Biocomposites for Antibacterial Coating. Polymers.

[65] Sabaruddin F.A., Paridah M.T., Sapuan S.M., Ilyas R.A., Lee S.H., Abdan K., Mazlan N., Roseley A.S.M., Abdul Khalil H.P.S. (2020). The effects of unbleached and bleached nanocellulose on the thermal and flammability of polypropylene-reinforced kenaf core hybrid polymer bionanocomposites. Polymers.

[66] Ilyas R.A., Sapuan S.M., Ibrahim R., Abral H., Ishak M.R., Zainudin E.S., Asrofi M., Atikah M.S.N., Huzaifah M.R.M., Radzi A.M. (2019). Sugar palm (Arenga pinnata (Wurmb.) Merr) cellulosic fibre hierarchy: A comprehensive approach from macro to nano scale. J. Mater. Res. Technol..

[67] Ilyas R.A., Sapuan S.M., Ishak M.R., Zainudin E.S. (2019). Sugar palm nanofibrillated cellulose (Arenga pinnata (Wurmb.) Merr): Effect of cycles on their yield, physic-chemical, morphological and thermal behavior. Int. J. Biol. Macromol..

[68] Ilyas R.A., Sapuan S.M., Ishak M.R., Zainudin E.S. (2018). Water transport properties of bio-nanocomposites reinforced by sugar palm (arenga pinnata) nanofibrillated cellulose. J. Adv. Res. Fluid Mech. Therm. Sci..

[69] Ilyas R.A., Sapuan S.M., Ishak M.R. (2018). Isolation and characterization of nanocrystalline cellulose from sugar palm fibres (Arenga Pinnata). Carbohydr. Polym..

[70] Ilyas R.A., Sapuan S.M., Ibrahim R., Abral H., Ishak M.R., Zainudin E.S., Atikah M.S.N., Mohd Nurazzi N., Atiqah A., Ansari M.N.M. (2019). Effect of sugar palm nanofibrillated celluloseconcentrations on morphological, mechanical andphysical properties of biodegradable films basedon agro-waste sugar palm (Arenga pinnata (Wurmb.) Merr) starch. J. Mater. Res. Technol..

[71] Ilyas R.A., Sapuan S.M., Atiqah A., Ibrahim R., Abral H., Ishak M.R., Zainudin E.S., Nurazzi N.M., Atikah M.S.N., Ansari M.N.M. (2020). Sugar palm (Arenga pinnata [Wurmb.] Merr) starch films containing sugar palm nanofibrillated cellulose as reinforcement: Water barrier properties. Polym. Compos..

[72] Abral H., Ariksa J., Mahardika M., Handayani D., Aminah I., Sandrawati N., Pratama A.B., Fajri N., Sapuan S.M., Ilyas R.A. (2020). Transparent and antimicrobial cellulose film from ginger nanofiber. Food Hydrocoll..

[73] Ilyas R.A., Sapuan S.M., Ibrahim R., Abral H., Ishak M.R., Zainudin E.S., Atiqah A., Atikah M.S.N., Syafri E., Asrofi M. (2020). Thermal, Biodegradability and Water Barrier Properties of Bio-Nanocomposites Based on Plasticised Sugar Palm Starch and Nanofibrillated Celluloses from Sugar Palm Fibres. J. Biobased Mater. Bioenergy.

[74] Ilyas R.A., Sapuan S.M., Sanyang M.L., Ishak M.R., Zainudin E.S. (2018). Nanocrystalline cellulose as reinforcement for polymeric matrix nanocomposites and its potential applications: A Review. Curr. Anal. Chem..

[75] Cosgrove D.J. (2005). Growth of the plant cell wall. Nat. Rev. Mol. Cell Biol..

[76] Martins M.A., Kiyohara P.K., Joekes I. (2004). Scanning electron microscopy study of raw and chemically modified sisal fibers. J. Appl. Polym. Sci..

[77] Ferreira F.V., Mariano M., Rabelo S.C., Gouveia R.F., Lona L.M.F. (2018). Isolation and surface modification of cellulose nanocrystals from sugarcane bagasse waste: From a micro- to a nano-scale view. Appl. Surf. Sci..

[78] Jarvis M.C. (2018). Structure of native cellulose microfibrils, the starting point for nanocellulose manufacture. Philos. Trans. R. Soc. A Math. Phys. Eng. Sci..

[79] Norrrahim M.N.F., Mohd Kasim N.A., Knight V.F., Abdul Halim N., Ahmad Shah N.A., Mohd Noor S.A., Jamal S.H., Ong K.K., Wan Yunus W.M.Z., Ahmad Farid M.A. (2021). Performance Evaluation of Cellulose Nanofiber Reinforced Polymer Composites. Funct. Compos. Struct..

[80] Aiza Jaafar C.N., Zainol I., Ishak N.S., Ilyas R.A., Sapuan S.M. (2021). Effects of the Liquid Natural Rubber (LNR) on Mechanical Properties and Microstructure of Epoxy/Silica/Kenaf Hybrid Composite for Potential Automotive Applications. J. Mater. Res. Technol..

[81] Abral H., Chairani M.K., Rizki M.D., Mahardika M., Handayani D., Sugiarti E., Muslimin A.N., Sapuan S.M., Ilyas R.A. (2021). Characterization of compressed bacterial cellulose nanopaper film after exposure to dry and humid conditions. J. Mater. Res. Technol..

[82] Omran A.A.B., Mohammed A.A.B.A., Sapuan S.M., Ilyas R.A., Asyraf M.R.M., Koloor S.S.R., Petrů M. (2021). Micro- and Nanocellulose in Polymer Composite Materials: A Review. Polymers.

[83] Aisyah H.A., Paridah M.T., Sapuan S.M., Ilyas R.A., Khalina A., Nurazzi N.M., Lee S.H., Lee C.H. (2021). A Comprehensive Review on Advanced Sustainable Woven Natural Fibre Polymer Composites. Polymers.

[84] Nurazzi N.M., Asyraf M.R.M., Khalina A., Abdullah N., Aisyah H.A., Rafiqah S.A., Sabaruddin F.A., Kamarudin S.H., Norrrahim M.N.F., Ilyas R.A. (2021). A Review on Natural Fiber Reinforced Polymer Composite for Bullet Proof and Ballistic Applications. Polymers.

[85] Abral H., Pratama A.B., Handayani D., Mahardika M., Aminah I., Sandrawati N., Sugiarti E., Muslimin A.N., Sapuan S.M., Ilyas R.A. (2021). Antimicrobial Edible Film Prepared from Bacterial Cellulose Nanofibers/Starch/Chitosan for a Food Packaging Alternative. Int. J. Polym. Sci..

[86] Alvarez V.A., Ruseckaite R.A., Vazquez A. (2003). Mechanical Properties and Water Absorption Behavior of Composites Made from a Biodegradable Matrix and Alkaline-Treated Sisal Fibers. J. Compos. Mater..

[87] Alemdar A., Sain M. (2008). Isolation and characterization of nanofibers from agricultural residues—Wheat straw and soy hulls. Bioresour. Technol..

[88] Julie Chandra C.S., George N., Narayanankutty S.K. (2016). Isolation and characterization of cellulose nanofibrils from arecanut husk fibre. Carbohydr. Polym..

[89] Chirayil C.J., Joy J., Mathew L., Mozetic M., Koetz J., Thomas S. (2014). Isolation and characterization of cellulose nanofibrils from Helicteres isora plant. Ind. Crops Prod..

[90] Cherian B.M., Leão A.L., de Souza S.F., Thomas S., Pothan L.A., Kottaisamy M. (2010). Isolation of nanocellulose from pineapple leaf fibres by steam explosion. Carbohydr. Polym..

[91] Syafri E., Kasim A., Abral H., Asben A. (2018). Cellulose nanofibers isolation and characterization from ramie using a chemical-ultrasonic treatment. J. Nat. Fibers.

[92] Megashah L.N., Ariffin H., Zakaria M.R., Hassan M.A. (2018). Properties of Cellulose Extract from Different Types of Oil Palm Biomass. IOP Conf. Ser. Mater. Sci. Eng..

[93] Jonoobi M., Khazaeian A., Tahir P.M., Azry S.S., Oksman K. (2011). Characteristics of cellulose nanofibers isolated from rubberwood and empty fruit bunches of oil palm using chemo-mechanical process. Cellulose.

[94] Corrêa A.C., de Morais Teixeira E., Pessan L.A., Mattoso L.H.C. (2010). Cellulose nanofibers from curaua fibers. Cellulose.

[95] Tibolla H., Pelissari F.M., Menegalli F.C. (2014). Cellulose nanofibers produced from banana peel by chemical and enzymatic treatment. LWT Food Sci. Technol..

[96] De Teixeira E.M., Bondancia T.J., Teodoro K.B.R., Corrêa A.C., Marconcini J.M., Mattoso L.H.C. (2011). Sugarcane bagasse whiskers: Extraction and characterizations. Ind. Crops Prod..

[97] Jonoobi M., Harun J., Shakeri A., Misra M., Oksmand K. (2009). Chemical composition, crystallinity, and thermal degradation of bleached and unbleached kenaf bast (Hibiscus cannabinus) pulp and nanofibers. BioResources.

[98] Bendahou A., Habibi Y., Kaddami H., Dufresne A. (2009). Physico-chemical characterization of palm from Phoenix Dactylifera-L, preparation of cellulose whiskers and natural rubber-based nanocomposites. J. Biobased Mater. Bioenergy.

[99] Chan C.H., Chia C.H., Zakaria S., Ahmad I., Dufresne A. (2013). Production and characterisation of cellulose and nano- crystalline cellulose from kenaf core wood. BioResources.

[100] Abral H., Dalimunthe M.H., Hartono J., Efendi R.P., Asrofi M., Sugiarti E., Sapuan S.M., Park J.W., Kim H.J. (2018). Characterization of Tapioca Starch Biopolymer Composites Reinforced with Micro Scale Water Hyacinth Fibers. Starch/Staerke.

[101] Alemdar A., Sain M. (2008). Biocomposites from wheat straw nanofibers: Morphology, thermal and mechanical properties. Compos. Sci. Technol..

[102] Li M., Wang L.J., Li D., Cheng Y.L., Adhikari B. (2014). Preparation and characterization of cellulose nanofibers from de-pectinated sugar beet pulp. Carbohydr. Polym..

[103] Sheltami R.M., Abdullah I., Ahmad I., Dufresne A., Kargarzadeh H. (2012). Extraction of cellulose nanocrystals from mengkuang leaves (Pandanus tectorius). Carbohydr. Polym..

[104] Hagstrand P.O., Bonjour F., Månson J.A.E. (2005). The influence of void content on the structural flexural performance of unidirectional glass fibre reinforced polypropylene composites. Compos. Part A Appl. Sci. Manuf..

[105] Huang H., Talreja R. (2005). Effects of void geometry on elastic properties of unidirectional fiber reinforced composites. Compos. Sci. Technol..

[106] Liebig W.V., Viets C., Schulte K., Fiedler B. (2015). Influence of voids on the compressive failure behaviour of fibrereinforced composites. Compos. Sci. Technol..

[107] Ruiz E., Achim V., Soukane S., Trochu F., Bréard J. (2006). Optimization of injection flow rate to minimize micro/macro-voids formation in resin transfer molded composites. Compos. Sci. Technol..

[108] Lukaszewicz D.H.J.A., Potter K.D. (2011). The internal structure and conformation of prepreg with respect to reliable automated processing. Compos. Part A Appl. Sci. Manuf..

[109] Liu L., Zhang B.M., Wang D.F., Wu Z.J. (2006). Effects of cure cycles on void content and mechanical properties of composite laminates. Compos. Struct..

[110] Dong C. (2016). Effects of Process-Induced Voids on the Properties of Fibre Reinforced Composites. J. Mater. Sci. Technol..

[111] Syafiq R., Sapuan S.M., Zuhri M.Y.M., Ilyas R.A., Nazrin A., Sherwani S.F.K., Khalina A. (2020). Antimicrobial activities of starch-based biopolymers and biocomposites incorporated with plant essential oils: A review. Polymers.

[112] Nazrin A., Sapuan S.M., Zuhri M.Y.M., Ilyas R.A., Syafiq R., Sherwani S.F.K. (2020). Nanocellulose Reinforced Thermoplastic Starch (TPS), Polylactic Acid (PLA), and Polybutylene Succinate (PBS) for Food Packaging Applications. Front. Chem..

[113] Asyraf M.R.M., Ishak M.R., Sapuan S.M., Yidris N., Ilyas R.A., Rafidah M., Razman M.R. (2020). Potential Application of Green Composites for Cross Arm Component in Transmission Tower: A Brief Review. Int. J. Polym. Sci..

[114] Asyraf M.R.M., Ishak M.R., Sapuan S.M., Yidris N., Shahroze R.M., Johari A.N., Rafidah M., Ilyas R.A. (2020). Creep test rig for cantilever beam: Fundamentals, prospects and present views. J. Mech. Eng. Sci..

[115] Azammi A.M.N., Ilyas R.A., Sapuan S.M., Ibrahim R., Atikah M.S.N., Asrofi M., Atiqah A. (2020). Characterization studies of biopolymeric matrix and cellulose fibres based composites related to functionalized fibre-matrix interface. Interfaces in Particle and Fibre Reinforced Composites.

[116] Sari N.H., Pruncu C.I., Sapuan S.M., Ilyas R.A., Catur A.D., Suteja S., Sutaryono Y.A., Pullen G. (2020). The effect of water immersion and fibre content on properties of corn husk fibres reinforced thermoset polyester composite. Polym. Test..

[117] Asrofi M., Sujito, Syafri E., Sapuan S.M., Ilyas R.A. (2020). Improvement of Biocomposite Properties Based Tapioca Starch and Sugarcane Bagasse Cellulose Nanofibers. Key Eng. Mater..

[118] Jumaidin R., Khiruddin M.A.A., Asyul Sutan Saidi Z., Salit M.S., Ilyas R.A. (2020). Effect of cogon grass fibre on the thermal, mechanical and biodegradation properties of thermoplastic cassava starch biocomposite. Int. J. Biol. Macromol..

[119] El-Shekeil Y.A., Sapuan S.M., Jawaid M., Al-Shuja’a O.M. (2014). Influence of fiber content on mechanical, morphological and thermal properties of kenaf fibers reinforced poly (vinyl chloride)/thermoplastic polyurethane poly-blend composites. Mater. Des..

[120] Özturk S. (2010). Effect of Fiber Loading on the Mechanical Properties of Kenaf and Fiberfrax Fiber-reinforced Phenol-Formaldehyde Composites. J. Compos. Mater..

[121] Xiao B., Huang Q., Chen H., Chen X., Long G. (2021). A fractal model for capillary flow through a single tortuous capillary with roughened surfaces in fibrous porous media. Fractals.

[122] Zakikhani P., Zahari R., Sultan M.T.H., Majid D.L. (2014). Extraction and preparation of bamboo fibre-reinforced composites. Mater. Des..

[123] Ramnath B.V., Manickavasagam V.M., Elanchezhian C., Krishna C.V., Karthik S., Saravanan K. (2014). Determination of mechanical properties of intra-layer abaca—jute—glass fiber reinforced composite. J. Mater..

[124] Al-Bahadly E.A.O. (2013). The Mechanical Properties of Natural Fiber Composites. Ph.D. Thesis.

[125] Karthi N., Kumaresan K., Sathish S., Gokulkumar S., Prabhu L., Vigneshkumar N. (2020). An overview: Natural fiber reinforced hybrid composites, chemical treatments and application areas. Mater. Today Proc..

[126] Boopalan M., Niranjanaa M., Umapathy M.J. (2013). Study on the mechanical properties and thermal properties of jute and banana fiber reinforced epoxy hybrid composites. Compos. Part B Eng..

[127] Sanjay M.R., Madhu P., Jawaid M., Senthamaraikannan P., Senthil S., Pradeep S. (2017). Characterization and Properties of Natural Fiber Polymer Composites: A Comprehensive Review. J. Clean. Prod..

[128] Kalaprasad G., Pradeep P., Mathew G., Pavithran C., Thomas S. (2000). Thermal conductivity and thermal diffusivity analyses of low-density polyethylene composites reinforced with sisal, glass and intimately mixed sisal/glass fibres. Compos. Sci. Technol..

[129] Mahjoub R., Yatim J.M., Mohd Sam A.R., Hashemi S.H. (2014). Tensile properties of kenaf fiber due to various conditions of chemical fiber surface modifications. Constr. Build. Mater..

[130] Brahmakumar M., Pavithran C., Pillai R.M. (2005). Science and Coconut fibre reinforced polyethylene composites: Effect of natural waxy surface layer of the fibre on fibre / matrix interfacial bonding and strength of composites. Compos. Sci. Technol..

[131] Baihaqi N.M.Z.N., Khalina A., Nurazzi N.M., Aisyah H.A., Sapuan S.M., Ilyas R.A. (2021). Effect of fiber content and their hybridization on bending and torsional strength of hybrid epoxy composites reinforced with carbon and sugar palm fibers. Polimery.

[132] Noorunnisa Khanam P., Abdul Khalil H.P.S., Jawaid M., Ramachandra Reddy G., Surya Narayana C., Venkata Naidu S. (2010). Sisal/Carbon Fibre Reinforced Hybrid Composites: Tensile, Flexural and Chemical Resistance Properties. J. Polym. Environ..

[133] Ramesh M., Palanikumar K., Reddy K.H. (2013). Mechanical property evaluation of sisal–jute–glass fiber reinforced polyester composites. Compos. Part B Eng..

[134] Kumar N.R., Prasad G.R., Rao B.R. (2012). Investigation on mechanical properties of short vakka fiber glass reinforced hybrid thermoplastic composites. Int. J. Eng. Res. Technol..

[135] Al-Maadeed M.A., Labidi S. (2014). Recycled polymers in natural fibre-reinforced polymer composites. Natural Fibre Composites.

[136] Shanmugam D., Thiruchitrambalam M. (2013). Static and dynamic mechanical properties of alkali treated unidirectional continuous Palmyra Palm Leaf Stalk Fiber/jute fiber reinforced hybrid polyester composites. Mater. Des..

[137] Srinivasan V., Rajendra Boopathy S., Sangeetha D., Vijaya Ramnath B. (2014). Evaluation of mechanical and thermal properties of banana–flax based natural fibre composite. Mater. Des..

[138] Asaithambi B., Ganesan G., Ananda Kumar S. (2014). Bio-composites: Development and mechanical characterization of banana/sisal fibre reinforced poly lactic acid (PLA) hybrid composites. Fibers Polym..

[139] Asaithambi B., Ganesan G.S., Ananda Kumar S. (2017). Banana/sisal fibers reinforced poly(lactic acid) hybrid biocomposites; influence of chemical modification of BSF towards thermal properties. Polym. Compos..

[140] Khan M.A., Ganster J., Fink H.-P. (2009). Hybrid composites of jute and man-made cellulose fibers with polypropylene by injection moulding. Compos. Part A Appl. Sci. Manuf..

[141] Dhakal H.N., Zhang Z.Y., Guthrie R., MacMullen J., Bennett N. (2013). Development of flax/carbon fibre hybrid composites for enhanced properties. Carbohydr. Polym..

[142] Ahmed K.S., Vijayarangan S. (2008). Tensile, flexural and interlaminar shear properties of woven jute and jute-glass fabric reinforced polyester composites. J. Mater. Process. Technol..

[143] Ramnath B.V., Kokan S.J., Raja R.N., Sathyanarayanan R., Elanchezhian C., Prasad A.R., Manickavasagam V.M. (2013). Evaluation of mechanical properties of abaca–jute–glass fibre reinforced epoxy composite. Mater. Des..

[144] Almeida J.H.S., Amico S.C., Botelho E.C., Amado F.D.R. (2013). Hybridization effect on the mechanical properties of curaua/glass fiber composites. Compos. Part B Eng..

[145] Zhang Y., Li Y., Ma H., Yu T. (2013). Tensile and interfacial properties of unidirectional flax/glass fiber reinforced hybrid composites. Compos. Sci. Technol..

[146] Akil H.M., Santulli C., Sarasini F., Tirillò J., Valente T. (2014). Environmental effects on the mechanical behaviour of pultruded jute/glass fibre-reinforced polyester hybrid composites. Compos. Sci. Technol..

[147] Atiqah A., Maleque M.A., Jawaid M., Iqbal M. (2014). Development of kenaf-glass reinforced unsaturated polyester hybrid composite for structural applications. Compos. Part B Eng..

[148] Singh B., Gupta M., Verma A. (1995). Mechanical behaviour of particulate hybrid composite laminates as potential building materials. Constr. Build. Mater..

[149] Arthanarieswaran V.P., Kumaravel A., Kathirselvam M. (2014). Evaluation of mechanical properties of banana and sisal fiber reinforced epoxy composites: Influence of glass fiber hybridization. Mater. Des..

[150] Yahaya R., Sapuan S.M., Jawaid M., Leman Z., Zainudin E.S. (2015). Effect of layering sequence and chemical treatment on the mechanical properties of woven kenaf–aramid hybrid laminated composites. Mater. Des..

[151] Yahaya R., Sapuan S.M., Leman Z., Zainudin E.S. (2014). Selection of Natural Fibre for Hybrid Laminated Composites Vehicle Spall Liners Using Analytical Hierarchy Process (AHP). Appl. Mech. Mater..

[152] Noorunnisa Khanam P., Abdul Khalil H.P.S., Ramachandra Reddy G., Venkata Naidu S. (2011). Tensile, Flexural and Chemical Resistance Properties of Sisal Fibre Reinforced Polymer Composites: Effect of Fibre Surface Treatment. J. Polym. Environ..

[153] Fiore V., Di Bella G., Valenza A. (2011). Glass–basalt/epoxy hybrid composites for marine applications. Mater. Des..

[154] Petrucci R., Santulli C., Puglia D., Sarasini F., Torre L., Kenny J.M. (2013). Mechanical characterisation of hybrid composite laminates based on basalt fibres in combination with flax, hemp and glass fibres manufactured by vacuum infusion. Mater. Des..

[155] Pavithran C., Mukherjee P.S., Brahmakumar M., Damodaran A.D. (1991). Impact properties of sisal-glass hybrid laminates. J. Mater. Sci..

[156] Yahaya R., Sapuan S.M., Jawaid M., Leman Z., Zainudin E.S. (2014). Mechanical performance of woven kenaf-Kevlar hybrid composites. J. Reinf. Plast. Compos..

[157] Yahaya R., Sapuan S.M., Jawaid M., Leman Z., Zainudin E.S. (2015). Effects of kenaf contents and fiber orientation on physical, mechanical, and morphological properties of hybrid laminated composites for vehicle spall liners. Polym. Compos..

[158] Narendar R., Priya Dasan K., Nair M. (2014). Development of coir pith/nylon fabric/epoxy hybrid composites: Mechanical and ageing studies. Mater. Des..

[159] Eesaee M., Shojaei A. (2014). Effect of nanoclays on the mechanical properties and durability of novolac phenolic resin/woven glass fiber composite at various chemical environments. Compos. Part A Appl. Sci. Manuf..

[160] Bledzki A.K., Franciszczak P., Meljon A. (2015). High performance hybrid PP and PLA biocomposites reinforced with short man-made cellulose fibres and softwood flour. Compos. Part A Appl. Sci. Manuf..

[161] Rout A.K., Satapathy A. (2012). Study on mechanical and tribo-performance of rice-husk filled glass–epoxy hybrid composites. Mater. Des..

[162] Arbelaiz A., Fernandez B., Cantero G., Llano-Ponte R., Valea A., Mondragon I. (2005). Mechanical properties of flax fibre/polypropylene composites. Influence of fibre/matrix modification and glass fibre hybridization. Compos. Part A Appl. Sci. Manuf..

[163] Reis P.N.B., Ferreira J.A.M., Antunes F.V., Costa J.D.M. (2007). Flexural behaviour of hybrid laminated composites. Compos. Part A Appl. Sci. Manuf..

[164] Yu X., Wei C., Lu S., Yu J., Xu D., Lu C. (2006). Preparation and mechanical properties of TLCP/ UP / GF in-situ hybrid composites. Trans. Nonferrous Met. Soc. China.

[165] Jawaid M., Abdul Khalil H.P.S., Abu Bakar A. (2010). Mechanical performance of oil palm empty fruit bunches/jute fibres reinforced epoxy hybrid composites. Mater. Sci. Eng. A.

[166] Jawaid M., Abdul Khalil H.P.S., Hassan A., Dungani R., Hadiyane A. (2013). Effect of jute fibre loading on tensile and dynamic mechanical properties of oil palm epoxy composites. Compos. Part B Eng..

[167] Santulli C., Sarasini F., Tirillò J., Valente T., Valente M., Caruso A.P., Infantino M., Nisini E., Minak G. (2013). Mechanical behaviour of jute cloth/wool felts hybrid laminates. Mater. Des..

[168] Alavudeen A., Rajini N., Karthikeyan S., Thiruchitrambalam M., Venkateshwaren N. (2015). Mechanical properties of banana/kenaf fiber-reinforced hybrid polyester composites: Effect of woven fabric and random orientation. Mater. Des..

[169] Venkateshwaran N., ElayaPerumal A., Alavudeen A., Thiruchitrambalam M. (2011). Mechanical and water absorption behaviour of banana/sisal reinforced hybrid composites. Mater. Des..

[170] Athijayamani A., Thiruchitrambalam M., Manikandan V., Pazhanivel B. (2010). Mechanical properties of natural fibers reinforced polyester hybrid composite. Int. J. Plast. Technol..

[171] Athijayamani A., Thiruchitrambalam M., Natarajan U., Pazhanivel B. (2009). Effect of moisture absorption on the mechanical properties of randomly oriented natural fibers/polyester hybrid composite. Mater. Sci. Eng..

[172] Venkateshwaran N., ElayaPerumal A. (2012). Mechanical and water absorption properties of woven jute/banana hybrid composites. Fibers Polym..

[173] Pérez-Fonseca A.A., Robledo-Ortíz J.R., Ramirez-Arreola D.E., Ortega-Gudiño P., Rodrigue D., González-Núñez R. (2014). Effect of hybridization on the physical and mechanical properties of high density polyethylene–(pine/agave) composites. Mater. Des..

[174] Graupner N., Herrmann A.S., Müssig J. (2009). Natural and man-made cellulose fibre-reinforced poly(lactic acid) (PLA) composites: An overview about mechanical characteristics and application areas. Compos. Part A Appl. Sci. Manuf..

[175] Suriani M.J., Sapuan S.M., Ruzaidi C.M., Naveen J., Syukriyah H. (2021). Correlation of manufacturing defects and impact behaviors of kenaf fiber reinforced hybrid fiberglass/Kevlar polyester composite. Polimery.

[176] Kaliappan P., Kesavan R., Ramnath B.V. (2016). Investigation on effect of fibre hybridization and orientation. Bull. Mater. Sci..

[177] Faur-Csukat G. (2007). Development of composite structures for ballistic protection. Mater. Sci. Forum.

[178] Huber T., Bickerton S., Müssig J., Pang S., Staiger M.P. (2012). Solvent infusion processing of all-cellulose composite materials. Carbohydr. Polym..

[179] Ibrahin N., Hadithon K., Abdan K. (2010). Effect of Fiber Treatment on Mechanical Properties of Kenaf Fiber—Ecoflex Composites. J. Reinf. Plast. Compos..

[180] Aslan M., Tufan M., Küçükömeroğlu T. (2018). Tribological and mechanical performance of sisal-filled waste carbon and glass fibre hybrid composites. Compos. Part B.

